# Reemergence of Murine Typhus in Galveston, Texas, USA, 2013

**DOI:** 10.3201/eid2103.140716

**Published:** 2015-03

**Authors:** Lucas S. Blanton, Rahat F. Vohra, Donald H. Bouyer, David H. Walker

**Affiliations:** University of Texas Medical Branch, Galveston, Texas, USA

**Keywords:** *Rickettsia typhi*, rickettsioses, murine typhus, rats, fleas, ectoparasites, vector-borne infections, vectorborne, flea-borne, fleaborne, *Xenopsylla cheopis*, Galveston, Texas, bacteria, rickettsia, ticks

## Abstract

Twelve patients with murine typhus were identified in Galveston, Texas, USA, in 2013. An isolate from 1 patient was confirmed to be *Rickettsia typhi*. Reemergence of murine typhus in Galveston emphasizes the importance of vector control and awareness of this disease by physicians and public health officials.

Murine typhus, caused by *Rickettsia typhi*, is typically transmitted to humans by *Xenopsylla cheopis*, a flea that infests rats. The disease is endemic to tropical and subtropical seaboard regions throughout the world (*1*). Before the introduction of DDT in 1946 as a means of controlling rat ectoparasites, murine typhus was a frequent cause of illness in Galveston, Texas, USA (*2*). After nearly 8 decades during which no cases were identified in this city (although unreported cases might have occurred), murine typhus was diagnosed in 2 Galveston residents in April (*3*) and October 2012 (L.S. Blanton, unpub. data). These cases prompted this study of murine typhus as a cause of undifferentiated febrile illness in residents of Galveston County who sought care at the University of Texas Medical Branch in Galveston.

## The Study

Physicians who were aware of the study alerted our team of investigators to patients >18 years of age who had reported fever during February–December 2013. Alternative syndromes that warranted exclusion included skin and soft tissue infections, urinary tract infections, cavitary or lobar pneumonia, and pyogenic intraabdominal processes (e.g., abscesses, appendicitis, cholecystitis, and diverticulitis). We obtained informed consent, medical history, and physical examination to record symptoms, signs, and laboratory data from each patient. We collected blood for use in real-time PCR, shell vial culture, and immunofluorescence assay (IFA) for the diagnosis of murine typhus (*4*–*6*). The conjugate used in IFA assays was Alexa Fluor 488 goat anti-human IgG (γ-chain–specific) at a dilution of 1:400 (Jackson ImmunoResearch Laboratories, West Grove, PA, USA). We made extensive attempts to collect blood from enrollees for convalescent IFA testing. Further PCR analysis to amplify portions of the 17-kDa antigen, citrate synthase, and outer membrane protein B (*ompB*) genes were performed on DNA from a cell culture isolate (*7*–*9*). Amplicons were cloned and sequenced (*4*).

A confirmed case of murine typhus was defined as having compatible signs and symptoms and seroconversion (diagnostic cutoff of 1:64) or a 4-fold increase in IgG titer against *R. typhi* from acute- and convalescent-phase serum samples; PCR detection of rickettsial DNA within acute-phase blood; or isolation of *R. typhi* from blood. A probable case was defined as having a single IgG titer of >1:256 during acute illness.

In addition to identifying acutely ill patients, we sought to determine the local prevalence of those who had *R. typhi* reactive antibodies. We repurposed serum samples collected from 500 Galveston residents; the samples were scheduled to be discarded after being used for routine clinical testing from outpatient clinics during the summer and fall of 2013. We screened for the presence of *R. typhi* IgG at a titer of 1:128 by IFA as described above. Endpoint titers were established in reactive samples. We performed IFA against spotted fever antigens (*R. rickettsii*) and Western blot analysis using *R. typhi* and *R. rickettsii* antigens to confirm the specificity of samples reactive to *R. typhi* as previously described (*6*,*10*). The University of Texas Medical Branch institutional review board approved these studies.

Eighteen patients who met study criteria were enrolled. Of these, 10 were determined to have murine typhus (7 confirmed and 3 probable) (Table 1). In addition to those identified prospectively, 2 probable cases were identified retrospectively. Serologic testing was the primary diagnostic method (6 of 7 confirmed cases). One case was confirmed by isolation of *R. typhi* from blood. This patient was an alcoholic man, 48 years of age, who sought care 4 days after the onset of fever, chills, and myalgias. Sequences of portions of the citrate synthase (GenBank accession no. KJ648945) and *ompB* (accession no. KJ648946) genes of this isolate revealed 100% homology to *R. typhi* Wilmington strain, and the sequence of the 17-kDa antigen gene (accession no. KJ648944) revealed 1 base pair difference. Real-time PCR screening of DNA extracted from whole blood demonstrated the presence of rickettsial DNA in the patient from whom the *R. typhi* isolate was obtained.

**Table 1:** Summary of reciprocal antibody titers in 12 patients with murine typhus, Galveston, Texas, 2013*

Patient no.	Illness duration†	Acute titer (date collected)	Convalescent titer (date collected)	Diagnosis by case definition
1	12 d	256 (Apr 10)	1,024 (Apr 30)	Confirmed
4‡	4 d	NR (May 23)	NA	Confirmed
6	4 d	NR (Jun 16)	128 (Jul 2)	Confirmed
8	7 d	NR (Jul 10)	512 (Aug 8)	Confirmed
9	10 d	NR (Jul 16)	256 (Jul 30)	Confirmed
10	13 d	NR (Jul 19)	256 (Jul 30)	Confirmed
17	6 d	NR (Sep 27)	1,024 (Dec 13)	Confirmed
13	8 d	256 (Jul 29)	NA	Probable
15	8 d	256 (Aug 9)	NA	Probable
16§	3 wk¶	256 (Jul 30)	NA	Probable
19	3 wk	512 (Oct 30)	NA	Probable
20§	8 d	256 (Mar 26)	NA	Probable

*NR, nonreactive at a titer of 1:64; NA, not available. †Duration of illness before workup and treatment. ‡Confirmed by culture isolate. §Case identified retrospectively. ¶Patient was treated with levofloxacin 2 weeks before murine typhus workup.

In addition to fever, the 12 patients, all reported headache, 7 (58%) had chills, 6 (50%) reported myalgias, 6 (50%) had rashes, and 9 (75%) had elevated hepatic transaminases (75%). Seven (58%) were hospitalized, and 2 (17%) were admitted to the intensive care unit. Except for 1 of the retrospectively identified cases, all patients were treated empirically for murine typhus (Table 1). Eleven (92%) were treated with either doxycycline or minocycline; minocycline was prescribed for some because of a national shortage of oral doxycycline. All patients had subsequent resolution of illness with no obvious difference in recovery between the 2 drugs. One patient (identified retrospectively) was treated with levofloxacin and reported slow resolution of her symptoms.

The 500 serum samples screened by IFA represent ≈1% of Galveston’s population (48,733 by the 2013 census [http://quickfacts.census.gov/qfd/states/48/4828068.html; cited 2014 Dec 27]). The mean age of patients whose samples were tested was 61.5 years. IFA and Western blot testing showed 8 (1.6%) samples to be reactive, which supported typhus group specificity (Table 2). Of those seroreactive, the mean age was 60.9 years, and 5 (62.5%) were women. The geometric mean reciprocal titer was 181.

**Table 2:** Analysis of serosurvey samples found reactive to *Rickettsia typhi* by IFA and Western blotting, Galveston, Texas, 2013*

Sample	Reciprocal IFA titers			Western blot analysis	
	*R. typhi*	*R. rickettsii*		*R. typhi* protein lysate (OmpB)†	*R. rickettsii* protein lysate (OmpA‡)§
1	256	NR		+	–¶
2	128	NR		+	–
3	128	NR		+	–
4	128	NR		+	–
5	256	NR		+	–
6	128	NR		+	–
7	1024	128		+	–
8	128	NR		+	–

*Omp, outer membrane protein; NR, nonreactive at a titer of 1:128. †Reactivity to the panrickettsial 135-kDa OmpB. Reactivity to the spotted fever group 190-kDa OmpA. §All serum samples reacted with *R. rickettsii* OmpB as described for spotted fever and typhus group OmpB antigens (*10*). ¶Bands corresponding to reactivity to OmpB but not OmpA confirms typhus group specificity.

## Conclusions

Galveston is a small city on a barrier island off the upper Texas coast, along the Gulf of Mexico. As with other port cities where rat population numbers are high, the incidence of murine typhus in this city was historically high. However, in 1946, as part of an ectoparasite eradication program, the insecticide DDT was applied to common rat paths; subsequently, the number of ectoparasites spread by rats in human habitats was reduced. As a result, the incidence of murine typhus in Galveston decreased dramatically (*2*) and continued to decline through subsequent decades (*11*). This study describes what is probably the reemergence of murine typhus in this area.

The patients in this study exhibited typical signs and symptoms consistent with murine typhus (*12*). Although we cannot exclude the possibility of a different fleaborne rickettsia (i.e., *R. felis*) infecting some patients, the single rickettsial isolate identified supports *R. typhi* as a causative agent. The seroprevalence supports the occurrence of additional undiagnosed cases. Based solely on the titers of these reactive serum samples, it is not possible to elucidate recent versus distant infections. Sporadic cases may have gone undiagnosed.

Dynamic shifts in the epidemiology and transmission of murine typhus are not unprecedented. Although the rat-to-rat cycle of transmission by fleas is often referred to as an urban cycle, the rural South experienced high rates of murine typhus in the 1940s as a result of a proliferation of rats after a change in crop production from cotton to peanuts, because rats were attracted to the peanuts as a source of food (*13*). In southern California, opossums infested with *R. typhi*– and *R. felis*–infected cat fleas (*C. felis*) have been associated with a shift of fleaborne rickettsioses from the urban center of Los Angles to suburban areas (*14*). This suburban cycle of transmission involving *C. felis* plays a recognized role in Corpus Christi, Texas, a coastal city ≈220 miles southwest of Galveston (*1*). Additionally, this cycle has been suspected in a recent outbreak of murine typhus in the central Texas city of Austin (*15*).

The recent recognition of murine typhus in Galveston may reflect the reemergence of *R. typhi* in rats; it may also reflect a cycle involving opossums and cats. Additionally, *R. felis* may play a role as a serologically cross-reacting culprit of illness. Further study is required to better understand the ecology and epidemiology of murine typhus as it reemerges in Galveston. Physicians and public health officials should be aware of this reemerging threat. Furthermore, vector control is of utmost importance.
